# Optimization of Metal-Assisted Chemical Etching for Deep Silicon Nanostructures

**DOI:** 10.3390/nano11112806

**Published:** 2021-10-22

**Authors:** Rabia Akan, Ulrich Vogt

**Affiliations:** KTH Royal Institute of Technology, Department of Applied Physics, Albanova University Center, 106 91 Stockholm, Sweden; ulrich.vogt@biox.kth.se

**Keywords:** metal-assisted chemical etching, Si nanostructures, high aspect ratio, zone plate

## Abstract

High-aspect ratio silicon (Si) nanostructures are important for many applications. Metal-assisted chemical etching (MACE) is a wet-chemical method used for the fabrication of nanostructured Si. Two main challenges exist with etching Si structures in the nanometer range with MACE: keeping mechanical stability at high aspect ratios and maintaining a vertical etching profile. In this work, we investigated the etching behavior of two zone plate catalyst designs in a systematic manner at four different MACE conditions as a function of mechanical stability and etching verticality. The zone plate catalyst designs served as models for Si nanostructures over a wide range of feature sizes ranging from 850 nm to 30 nm at 1:1 line-to-space ratio. The first design was a grid-like, interconnected catalyst (brick wall) and the second design was a hybrid catalyst that was partly isolated, partly interconnected (fishbone). Results showed that the brick wall design was mechanically stable up to an aspect ratio of 30:1 with vertical Si structures at most investigated conditions. The fishbone design showed higher mechanical stability thanks to the Si backbone in the design, but on the other hand required careful control of the reaction kinetics for etching verticality. The influence of MACE reaction kinetics was identified by lowering the oxidant concentration, lowering the processing temperature and by isopropanol addition. We report an optimized MACE condition to achieve an aspect ratio of at least 100:1 at room temperature processing by incorporating isopropanol in the etching solution.

## 1. Introduction

Nanostructured Si is the material of choice for a variety of applications such as photonics [1], lithium ion batteries [2], solar cells [3], biosensors [4], microfluidic channels [5] and X-ray optics [6]. Many of these applications require devices with highly vertical and deep Si nanostructures, i.e., high aspect ratios. The smaller the structures are, the more challenging it gets to fabricate high-aspect ratio nanostructures that are mechanically stable.

Both dry and wet etching processes are used for the fabrication of Si devices. Reactive ion etching (RIE) [7] is an example of a commonly used dry etching technique. For Si devices with nanostructures, maintaining a vertical etching profile becomes challenging with RIE and thus limits the achievable aspect-ratios [8]. Therefore, RIE is more suitable for the fabrication of devices with structures in the micrometer range or when extreme aspect ratios are not needed.

A wet etching process that overcomes fabrication challenges is MACE. MACE is an electroless method that is gaining a lot of attention as a pattern transfer technique for the fabrication of deep high-aspect ratio Si nanostructures [9,10]. In MACE, a noble metal catalyst (e.g., gold (Au) that is lithographically defined or in nanoparticle form) is etching its way into a Si substrate in an electrolyte (etching solution) composed of hydrofluoric acid (HF) and a strong oxidizer (e.g., hydrogen peroxide (H${}_{2}$O${}_{2}$)). The noble metal (cathode) catalyzes the reduction of the oxidizer and consequently electrical holes are formed. The holes are injected into the Si substrate (anode) locally on the noble metal site and oxidizes the substrate. The HF then dissolves the oxidized Si and the catalyst pattern is transferred into the Si substrate.

Although conceptually simple, MACE can become complicated since many parameters influence the process like etching solution composition [11], etching temperature [12], doping concentration of the Si substrate [13,14], catalyst thickness [15] or catalyst morphology [16,17]. These parameters have to be carefully adjusted to obtain the desired etching performance. In the literature, there are numerous studies investigating many of these parameters and their effects on micro- and nanostructure etching. However, comparing results is often difficult due to the variation in active etching areas and structure sizes. There is a lack of systematic studies that make direct comparison of etching behavior possible.

For etching of catalyst structures in the nanometer range two main challenges exist. Firstly, maintaining a vertical etching direction and secondly, keeping a good mechanical stability for deep etching when reaching extreme aspect ratios. Especially, the catalyst design has a great impact on these two challenges [11]. In this work we choose zone plate structures as model catalyst patterns to find the morphological parameters and etching conditions that are best suited for MACE processing of sub-100 nm high-aspect ratio Si structures.

Zone plates are diffractive imaging and focusing optics commonly used in X-ray microscopes [18]. Their circular grating structures are decreasing in width with the zone plate radii. Two parameters are key for the zone plate performance: imaging resolution and diffraction efficiency. The zone plate resolution is defined by the outermost zone width, whereas the diffraction efficiency is defined by the zone thickness [19]. The X-ray energy and zone plate material will define the required zone thickness. For the use in the hard X-ray regime, thicknesses of several micrometers are often needed. In order to fabricate a high-resolution and high-efficient zone plate, very high aspect ratios are therefore required. Since X-ray zone plates contain structures ranging from micron-sized features in the center to nanometer-sized features in the outer parts, they are ideal model patterns to systematically investigate the MACE process. The obtained results are applicable to other Si devices with nanostructured, lithographically defined catalyst patterns.

**Figure 1:**
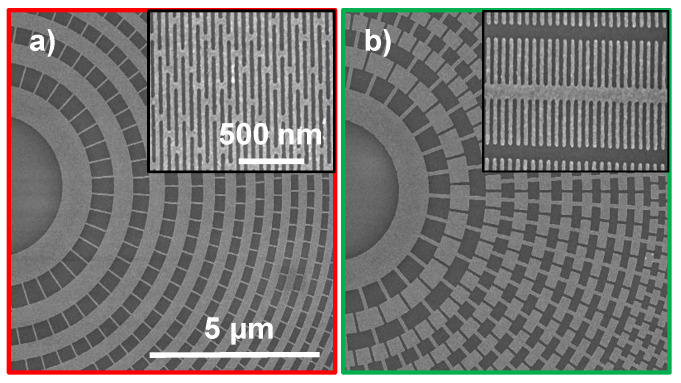
Top-view SEM micrographs of Au zone plate catalyst patterns on Si illustrating (**a**) brick wall (red frame) and (**b**) fishbone designs (green frame). The insets show the outermost parts of the zone plate designs. The zone plates designs have a 150 μm diameter, 1:1 line-to-space ratio and the outermost zone widths are 30 nm. Same scale bars apply to the micrographs.

We systematically investigate MACE of two catalyst pattern designs at four different MACE conditions in order to find optimum process conditions for obtaining vertical, mechanically stable deep high-aspect ratio Si nanostructures. The first design, called “brick wall” (Figure 1a), is a grid catalyst with all zones interconnected, whereas the second design, called “fishbone” (Figure 1b) is a hybrid catalyst with partly interconnected and partly isolated zones. Both designs have smallest feature sizes of 30 nm (width of the outermost zones), which are as far as we are aware the smallest lithographically defined catalyst structures reported for MACE. To our knowledge, this is the first study that combines the brick wall and fishbone type catalyst designs on the same chip, using the same active catalyst area and exposes them to the same exact reaction conditions, including cold etching and IPA addition. This makes direct comparisons of etching behavior possible.

## 2. Motivation for the Selection of Catalyst Designs and MACE Conditions

In the literature, there are several studies reporting the fabrication of Si zone plate nanostructures using MACE [11,20,21,22,23,24]. None of the reported studies contain a detailed motivation for their choice of catalyst design and MACE processing conditions, instead, the MACE pattern transfer has been presented as one in a series of steps for a complete device fabrication. Some of the studies used grid catalyst designs [11,20,24] while others preferred fishbone catalyst designs [21,22,23,25]. It has to be noted that it is difficult to etch isolated catalyst structures in a controlled way, so some kind of connected catalyst design is necessary in order to achieve vertical etching.

There are benefits and challenges with both grid and fishbone designs. A grid catalyst design has the advantage of maintaining a vertical etching profile owing to the interconnected zones, however, resulting in isolated Si nanostructures. The isolated Si structures will limit the achievable etch depths due to collapse, especially in smallest features [20]. In the fishbone design, the noble metal rings are interrupted forming sections with perpendicular lines crossing each section, resulting in partly interconnected Si after MACE. This interconnection will contribute with mechanical stability, but at the expense of etching verticality. It was recently reported that the fishbone design deviates from its vertical etching path at thicknesses beyond 1 μm due to the free Au ends in the design [23]. It should be noted that the results were obtained for a specific MACE processing condition, and it is therefore not possible to identify if this behavior is due to the catalyst design or the reaction conditions. Here, our aim is to directly compare these two types of catalyst designs by processing them on the same substrate and studying their etching behavior at different MACE conditions.

We base our choices of different MACE conditions on reaction kinetics. We want to control the hole injection rate into the Si, and thus, the overall etching rate by lowering the processing temperature and H${}_{2}$O${}_{2}$ concentration from our previously optimized MACE condition [11]. In our previous study, a grid catalyst design was used and the MACE condition was optimized based on etch depth and silicon zone roughness. The aim here is to investigate if a slower MACE reaction is beneficial for the etching directionality, as cold etching was used in several other studies without further explanation but showing nice vertical structures [21,22,23]. We further investigate the effect of isopropanol (IPA) which should also lower the etch rate [26]. Additionally, previous work suggests that the transport of generated gas from the etching location will be improved with the addition of IPA due to lower surface tension [27]. All MACE conditions are studied for different etching times to gain an understanding of to what etch depths the two designs are mechanically stable and if the different reaction conditions affect the mechanical stability of the zones. Our goal is to provide benchmark results for researchers in various fields that want to find optimum MACE processes for Si nanostructure fabrication.

## 3. Materials and Methods

**Figure 2:**
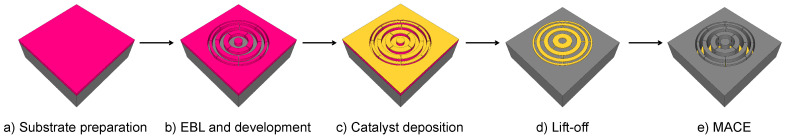
Overview of the experimental procedure. (**a**) Si substrate (grey) preparation by spin-coating 80 nm CSAR 62 resist (pink). (**b**) Zone plate patterning by EBL and development for removal of resist residues. (**c**) Deposition of 2 nm Ti and 10 nm Au (yellow) by evaporation. (**d**) Resist lift-off, leaving the metal zone plate pattern. (**e**) Etching the metal zone plate pattern into the Si via MACE.

Si p-type (100) wafers with resistivity of 1–5 Ω·cm were used for all samples. The zone plate fabrication procedure consisted of a sequence of steps: (1) Ultrasonic cleaning of wafer pieces followed by a oxygen plasma cleaning step, (2) resist spinning and patterning via electron-beam lithography, (3) resist development, (4) short oxygen plasma treatment, (5) electron beam evaporation of catalyst layer, (6) resist lift-off, (7) oxygen plasma cleaning for removal of organics, (8) MACE and (9) critical point drying. An overview of the experimental procedure is presented in Figure 2.

The Si wafers were cut into 1.5 cm × 1.5 cm chips and cleaned ultrasonically in acetone and IPA for removal of Si dust and by an oxygen plasma step (PlasmaLab 80 Plus RIE/ICP system, Oxford Instruments, Abingdon, UK) for removal of organics and further oxidising the Si surface (each cleaning step was typically 5 min). We used 80 nm of the positive resist CSAR 62 (Allresist GmbH, Strausberg, Germany) for the electron beam lithography step (50 kV Voyager EBL system, Raith GmbH, Dortmund, Germany) and the zone plate catalyst designs investigated in this study (brick wall and fishbone) were patterned on each substrate. The zone plate designs had a diameter of 150 μm, 1:1 line-to-space ratio and zone widths ranging from 850 nm (innermost zones) to 30 nm (outermost zones). The interconnects in the brick wall design were 40 nm and brick length of the outermost zones were 460 nm. In the fishbone design, the backbone was 95 nm and free ends were 360 nm of the outermost zones. The resist development was performed in amyl acetate (60 s) followed by rinsing in IPA and n-pentane (10 and 15 s, respectively). The short oxygen plasma treatment step (13 s) after development was to ensure removal of any resist residues in the zone plate patterns. For the catalyst layer, 10 nm Au was electron beam evaporated (in-house Eurovac/Thermionics deposition system) at 1 Å/s on a 2 nm adhesive Ti layer. The resist lift-off was performed in dimethyl succinate under ultrasonication. The final oxygen plasma step (3 min) was necessary to remove any organics on the sample surfaces that might prevent the MACE process.

The MACE experiments were all performed in a polytetrafluoroethylene (PTFE) container under light protection. Four different MACE conditions were used to investigate the etching behavior of the two designs (Table 1).

To understand the reproducibility of the MACE process, eight substrates with three zone plates per design were processed and each MACE condition was repeated twice. The etching solution compositions were based on previously reported studies [11,27]. The cold MACE experiments at 8 °C (C2) were performed using pre-cooled chemicals in a surrounding cooling bath. Both the patterned Si chips and etching solutions were kept in the cooling bath prior MACE to ensure temperature stabilization. To carefully investigate the etching direction and mechanical stability at different etch depths, the processing time was set to 3, 6, 9 and 12 min for each MACE condition. The samples were transferred to ethanol after MACE processing and further critical point dried (Leica EM CPD300, Leica Microsystems GmbH, Wetzlar, Germany). For etch depth analysis, cross-sections were prepared using FIB milling (Nova 200 NanoLab system, FEI Company, Hillsboro, OR, USA). Additionally, some samples were cleaved manually for a better visualization of cross sections. Unfortunately, this is a complicated procedure and cleaved cross-sections could not be obtained for all etching conditions and designs.

## 4. Results and Discussion

We characterize the etching behavior of our catalyst designs based on mechanical stability and etching verticality. The MACE conditions that are favorable for each design in terms of these two characteristics are identified and discussed.

### 4.1. Mechanical Stability

As previously reported, a grid-like catalyst design resulted in vertically etched Si structures, almost independently of the used MACE condition [11]. While its strength lay in maintaining etching verticality, the mechanical stability of the remaining Si structures became a limitation at too small structural widths and large etch depths. Zone plates were therefore ideal catalyst structures to identify at what point the mechanical instability started thanks to their broad size range of zones.

**Figure 3:**
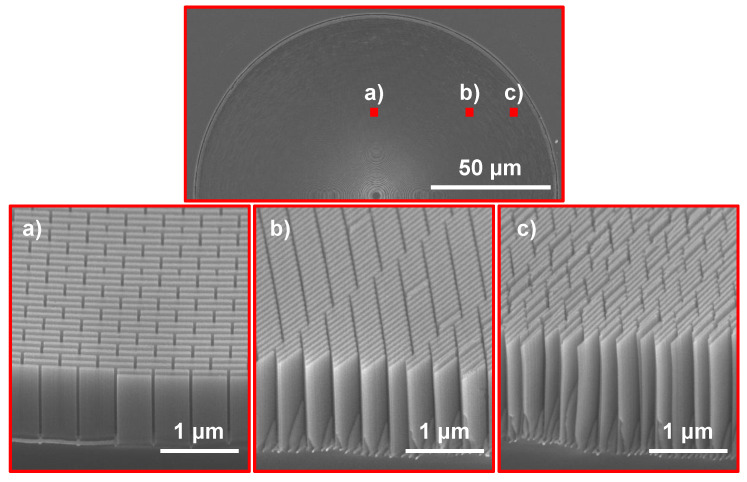
Brick wall catalyst design processed at MACE condition C1. The top micrograph shows where the three micrographs labeled (**a**–**c**) are localized on the zone plate. The cross-sections were prepared by manual cleaving.

Figure 3 shows cross-section micrographs of a ≈ 1.4 μm (average thickness over the zone plate radius) thick brick wall zone plate at three points with different zone widths. The zone width in Figure 3a was 60 nm and no collapse of zones were apparent. At 50 nm zone width, as shown in Figure 3b, some tendency of collapse was starting to show suggesting that a stability limit for the etch depth was reached at this point. At the outermost parts of the zone plate where the zone widths were 30 nm most of the Si nanostructures had collapsed (Figure 3c). It should be noted that the etch depth varied over the zone plate, where the innermost zones were less deep than the outermost parts. The exact depth where the collapse started appearing at 50 nm zone width was 1.5 μm, suggesting that the achievable aspect ratio of a stable brick wall design was 30:1.

**Figure 4:**
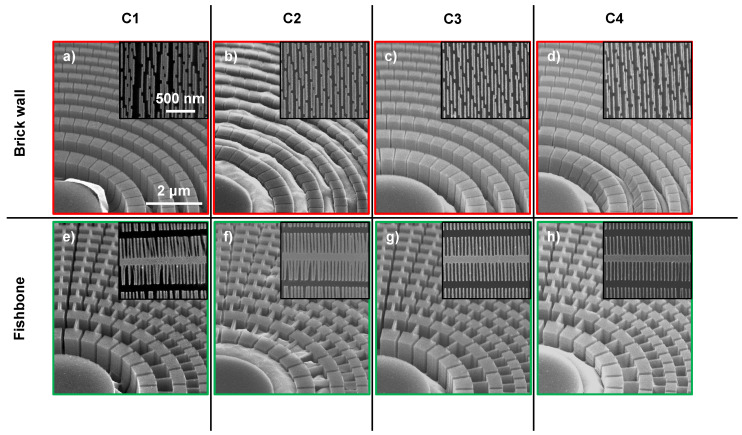
Brick wall (red frame) and fishbone (green frame) zone plate designs etched for 6 min at MACE conditions (**a**) C1, (**b**) C2, (**c**) C3 and (**d**) C4 and (**e**) C1, (**f**) C2, (**g**) C3 and (**h**) C4, respectively. The two designs shown per MACE condition were processed on the same Si chip. Same scale bars apply to main SEM micrographs and the insets. The insets show the outermost zones.

The etching behavior of the catalysts at the investigated conditions C1–C4 are summarized in Figure 4, a common etching time point of 6 min was chosen for comparison. The average etch rates for the different conditions are presented in Table 2. Room temperature processing with the higher H${}_{2}$O${}_{2}$ concentration gave the fastest etch rate (C1) which, based on the well accepted MACE theory [9,28], suggested the highest hole injection rate into the Si. The deepest etching of ≈2.4 μm was obtained at this condition and especially the outermost zones of both the brick wall (Figure 4a) and the fishbone (Figure 4e) design showed deformation.

**Figure 5:**
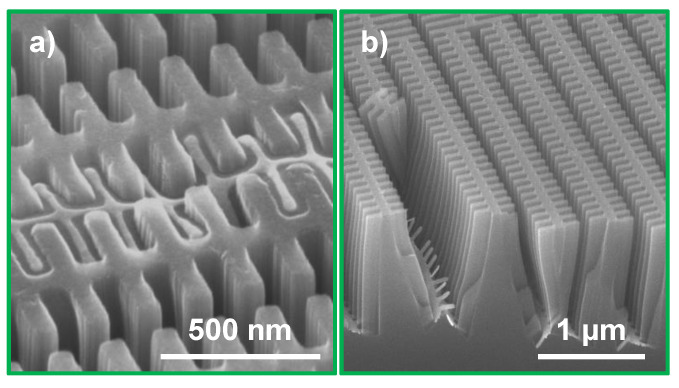
Fishbone zone plate catalyst etched at MACE condition C2. SEM micrographs show (**a**) local etching differences (6 min MACE) and (**b**) non-vertical etching (12 min MACE). The cross-section in (**b**) was prepared by manual cleaving.

Interestingly, a 10 times lower H${}_{2}$O${}_{2}$ concentration (C2), and consequently a slower etch rate (Table 2), did not result in a homogeneous and controlled etching. An uneven etching with both catalyst designs was more apparent here than with any other investigated MACE condition (Figure 4b,f). Especially for the fishbone design, local differences in etching within neighboring structures was observed suggesting an uneven H${}_{2}$O${}_{2}$ distribution over the catalyst area (see Figure 5a). The etch depth was ≈0.5 μm and surprisingly, at this shallow etch depth collapse of the outermost zone plate structures was observed for the brick wall catalyst (Figure 4b). A homogeneous and vertical etching was thus key for mechanical stability of the Si structures. Furthermore, these results showed that verticality was a prerequisite for the stability of the Si structures. It should be noted that we did not implement any stirring of the etching solution during MACE to avoid any disturbance of the process.

Lowering the processing temperature (C3) and adding IPA (C4) to the etching solution resulted as expected in slower etch rates [12,24,29] (Table 2). At etch depths of ≈1.7 μm and ≈1.4 μm with MACE conditions C3 and C4, respectively, a positive impact on the overall etching uniformity of both the brick wall (Figure 4c,d) and the fishbone (Figure 4g,h) catalysts was observed. With the IPA addition, slight broadening of the innermost Si structures could be seen (Figure 4d), but nonetheless, both conditions contributed to a better mechanical stability of the outermost structures.

### 4.2. Etching Verticality

**Figure 6:**
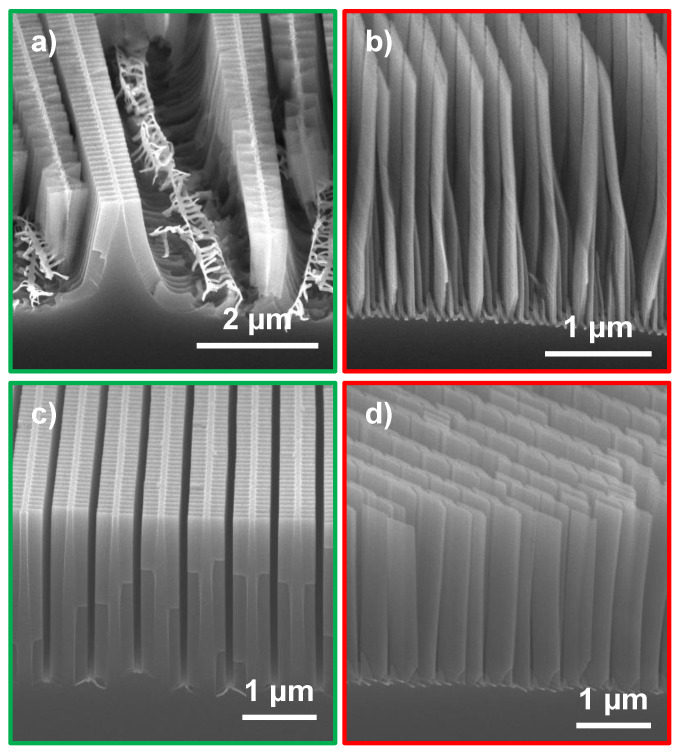
The effect of IPA on etching verticality. (**a**) fishbone and (**b**) brick wall catalyst designs etched at condition C1 (no IPA, same Si chip), and (**c**) fishbone and (**d**) brick wall catalysts etched at condition C4 (with IPA, same Si chip). The MACE time was 9 min for both samples. The cross-sections were prepared by cleaving.

Maintaining a vertical etch direction for isolated catalyst structures of nanometer sizes is challenging [30,31]. The fishbone design with partly isolated structures was also found to require more careful control of the MACE chemistry and reaction kinetics to maintain a vertical etching direction throughout the process. At the highest investigated etch rate (C1) deformation of the outermost zones of the fishbone catalyst indicated a deviated etch direction (Figure 4e). This was confirmed from the cross-section micrograph in Figure 6a, where the non-vertical etch profile as well as catalyst deformation is shown (≈2 μm etch depth). The brick wall design processed on the same Si chip maintained a vertical but collapsed etching profile (≈3 μm etch depth, Figure 6b). Similarly to the negative effect on the Si structure stability, the uneven or limited access of H${}_{2}$O${}_{2}$ to the catalyst surface also negatively impacted the etching verticality, although etch rates were significantly lowered. This is illustrated in Figure 4f (outermost zones) and Figure 5b.

Compared to conditions C1 and C2, conditions C3 and C4 indicated a more even etching, especially at the outermost parts of the fishbone catalyst design (Figure 4g,h). The impact IPA addition (C4) had on the etching verticality is illustrated in Figure 6c, where the zones are vertical and the etching profile is uniform. As for most other conditions, the brick wall catalyst processed on the same chip also kept a vertical etching profile at C4 (Figure 6d). The etch depth of both zone plate designs in Figure 6c,d was ≈3 μm. This suggests that aspect ratios of at least 100:1 with a maintained vertical etching profile that can be obtained by IPA addition.

**Figure 7:**
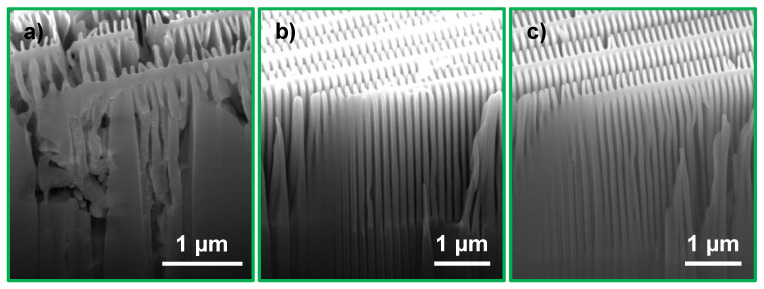
Cross-section SEM micrographs of the fishbone design catalyst processed at MACE condition (**a**) C1 for 9 min, (**b**) C3 for 12 min and (**c**) C4 for 12 min. The cross-sections were made by FIB milling.

Figure 7 shows micrographs of cross-sections prepared by FIB milling comparing the original MACE condition (C1, Figure 7a) with the relatively low processing temperature (C3, Figure 7b) and the addition of IPA (C4, Figure 7c) at average etch depths of ≈4.2 μm, ≈3.2 μm and ≈4 μm, respectively. It should be noted that FIB milling caused deformation of the finer structures, but still could give an indication of the etching directionality and depth. From these micrographs, it is obvious that both C3 and C4 were beneficial with regards to the etching direction. We draw the conclusion that controlling the MACE kinetics by lower processing temperature or by addition of IPA in the etching solution had similar, positive impacts on the etching verticality. However, the effect of IPA addition on the MACE process was not completely clear. If the effect of IPA was limited to reduction of the etch rate or if other mechanisms like easier release of formed gases were important is an open question. From a process handling point of view, we recommend adding IPA to the etching solution instead of temperature regulation of the etching solution. Temperature stabilization using a large volume of etching solution was time consuming and needed pre-cooling of the chemicals. The low heat conductivity of the PTFE container was altering the temperature of the chemicals and limited the number of MACE experiments that could be performed due to long temperature stabilization times in between samples. Therefore, to avoid uneven etch rates due to temperature elevation, room temperature processing with IPA is preferable.

### 4.3. Process Reproducibility

Many reports on MACE either use the process as one in a series of steps for micro- and nanofabrication, or investigate the process performance for a given application. As far as we are aware, there are no reports that discuss the reproducibility of the process. In this study, we explored four different MACE conditions and examined the etching behavior at four time points per condition, which in turn was repeated twice. We believe this large set of experiments gave a good indication of the reproducibility of the MACE process.

**Figure 8:**
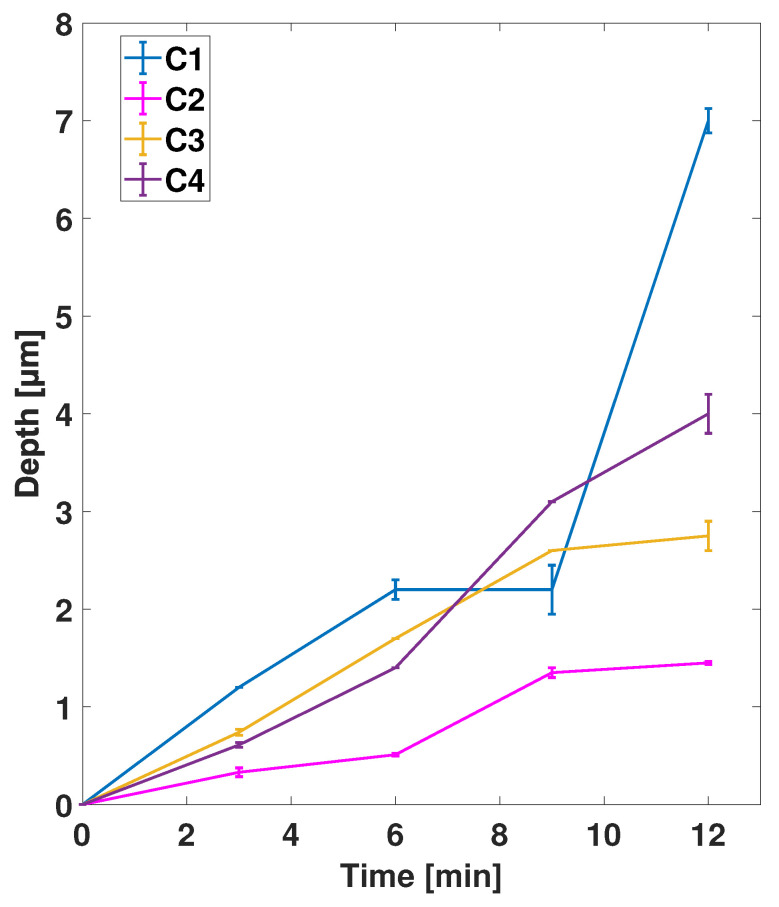
Average etch depths of brick wall and fishbone catalysts processed on the same Si chip as a function of etching time at MACE conditions C1–C4. The error bars show the standard deviation of the measurements.

Figure 8 shows the average etch depth of one brick wall and one fishbone zone plate catalyst design processed on the same chip as a function of processing time at conditions C1–C4. The error bars represent the standard deviation and indicate the variation in depth between the two zone plates. As previously reported, the decreasing width of the zones over the zone plate radius resulted in relatively deeper etching of the smallest, outermost zones than the largest, innermost zones. This gave a local variation of a few per cent over the same zone plate [11,20]. This variation is not shown here, but was present for all devices. Overall, all conditions gave larger zone thicknesses with longer processing time. However, the etch depth did not increase linearly with time. Even though both zone plates had the same active catalyst area, etch depth variations could be observed for certain time points.

**Figure 9:**
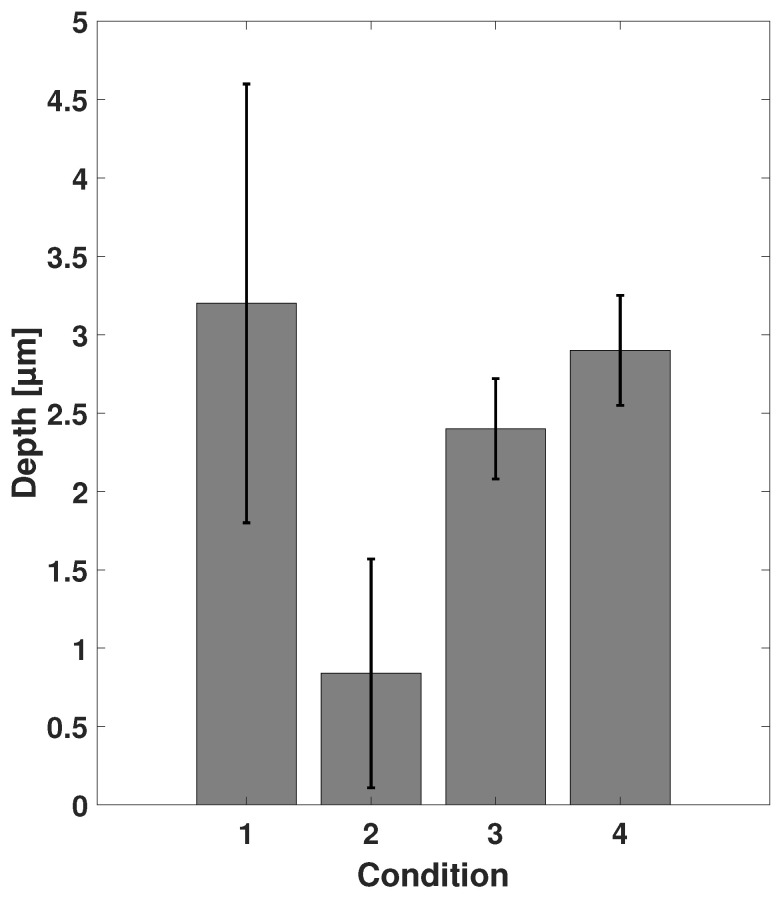
Average etch depths of zone plates on different Si chips processed at conditions C1–C4 for 9 min. The error bars indicate the depth variation between different chips.

Furthermore, differences in etch depths of zone plates on two different Si chips processed at identical conditions are shown in Figure 9. The error bars represent the standard deviation of the measurements. The variations in etch depth were largest for samples processed at conditions C1 and C2, and smaller for C3 and C4. Even though identical samples with same sizes and same active catalyst areas were processed at identical conditions, etching behavior differed depending on condition.

With these results, we want to highlight that precise control of the etching process was challenging and that the depth might vary not only between samples from run to run, but between structures on the same Si chip. The outcome of our experiments indicated that conditions C3 and C4 were also preferable for good reproducibility.

## 5. Conclusions

We demonstrated the MACE effect of catalyst design and reaction kinetics on fabrication of high-aspect ratio Si structures as a function of mechanical stability and etching verticality. Two zone plate catalyst designs with structural sizes ranging from 850 nm down to 30 nm have been used, brick wall (interconnected) and fishbone (partly connected and partly isolated), and their etching behavior at four different conditions has been systematically studied. Our findings reveal that the Si structures of the brick wall design were mechanically stable up to an aspect ratio of 30:1, while deeper etching resulted in collapse of the outermost zones with the smallest structures. Furthermore, etching verticality could be maintained with the brick wall design, independently of the MACE condition. The fishbone design required more careful control of the reaction kinetics for the catalyst to translate linearly into the Si. Addition of IPA and a lowered processing temperature showed a significant improvement in etching verticality of the fishbone design as opposed to room temperature processing without any additive in the etching solution. The cold MACE required a rather complicated cooling setup, and therefore, we recommend IPA addition to the etching solution for controlled kinetics at room temperature processing. For the future, the influence of other alcohols on the processing of Si nanostructures should be investigated. We also show data indicating the sensitivity of the MACE process. These are the first results showing the reproducibility of the process and more statistics are needed for further characterization.

We think our findings provide relevant information for micro- and nanofabrication of high-aspect ratio Si structures with MACE. The easy processing without any need of complicated experimental setups or tools will be beneficial for applications that require controlled Si fabrication with lithographically defined morphology.

## Figures and Tables

**Table 1:** Investigated MACE conditions.

Condition	[H${}_{2}$O${}_{2}$] : [HF] : [IPA] (M)	Temperature (°C)
C1	0.68 : 4.7 : 0	25
C2	0.068 : 4.7 : 0	25
C3	0.68 : 4.7 : 0	8
C4	0.68 : 4.7 : 0.91	25

**Table 2:** Average etch rates for the investigated MACE conditions.

Condition		Rate [μm/min]
C1		0.45
C2		0.090
C3		0.26
C4		0.28

## Data Availability

The data presented in this study are available on request from the corresponding author.

## References

[1] Karabchevsky A., Katiyi A., Ang A.S., Hazan A. (2020). On-chip nanophotonics and future challenges. Nanophotonics.

[2] Yang Y., Yuan W., Kang W., Ye Y., Pan Q., Zhang X., Ke Y., Wang C., Qiu Z., Tang Y. (2020). A review on silicon nanowire-based anodes for next-generation high-performance lithium-ion batteries from a material-based perspective. Sustain. Energy Fuels.

[3] Sahoo M.K., Kale P. (2019). Integration of silicon nanowires in solar cell structure for efficiency enhancement: A review. J. Mater..

[4] Xu Y., Hu X., Kundu S., Nag A., Afsarimanesh N., Sapra S., Mukhopadhyay S.C., Han T. (2019). Silicon-Based Sensors for Biomedical Applications: A Review. Sensors.

[5] Gale B., Jafek A., Lambert C., Goenner B., Moghimifam H., Nze U., Kamarapu S. (2018). A Review of Current Methods in Microfluidic Device Fabrication and Future Commercialization Prospects. Inventions.

[6] Romano L., Stampanoni M. (2020). Microfabrication of X-ray Optics by Metal Assisted Chemical Etching: A Review. Micromachines.

[7] Wu B., Kumar A., Pamarthy S. (2010). High aspect ratio silicon etch: A review. J. Appl. Phys..

[8] Huff M. (2021). Recent Advances in Reactive Ion Etching and Applications of High-Aspect-Ratio Microfabrication. Micromachines.

[9] Huang Z., Geyer N., Werner P., De Boor J., Gösele U. (2011). Metal-assisted chemical etching of silicon: A review. Adv. Mater..

[10] Han H., Huang Z., Lee W. (2014). Metal-assisted chemical etching of silicon and nanotechnology applications. Nano Today.

[11] Akan R., Parfeniukas K., Vogt C., Toprak M.S., Vogt U. (2018). Reaction control of metal-assisted chemical etching for silicon-based zone plate nanostructures. RSC Adv..

[12] Backes A., Leitgeb M., Bittner A., Schmid U. (2016). Temperature Dependent Pore Formation in Metal Assisted Chemical Etching of Silicon. ECS J. Solid State Sci. Technol..

[13] Geyer N., Wollschläger N., Fuhrmann B., Tonkikh A., Berger A., Werner P., Jungmann M., Krause-Rehberg R., Leipner H.S. (2015). Influence of the doping level on the porosity of silicon nanowires prepared by metal-assisted chemical etching. Nanotechnology.

[14] Aca-López V., Quiroga-González E., Gómez-Barojas E., Światowska J., Luna-López J.A. (2020). Effects of the doping level in the production of silicon nanowalls by metal assisted chemical etching. Mater. Sci. Semicond. Process..

[15] Um H.D., Kim N., Lee K., Hwang I., Hoon Seo J., Yu Y.J., Duane P., Wober M., Seo K. (2015). Versatile control of metal-assisted chemical etching for vertical silicon microwire arrays and their photovoltaic applications. Sci. Rep..

[16] Hildreth O.J., Lin W., Wong C.P. (2009). Effect of Catalyst Shape and Etchant Composition on Etching Direction in Metal-Assisted Chemical Etching of Silicon to Fabricate 3D Nanostructures. ACS Nano.

[17] Xia W., Zhu J., Wang H., Zeng X. (2014). Effect of catalyst shape on etching orientation in metal-assisted chemical etching of silicon. CrystEngComm.

[18] Attwood D., Sakdinawat A. (2016). X-Rays and Extreme Ultraviolet Radiation.

[19] Lider V.V. (2017). Zone Plates for X-Ray Focusing (Review). J. Surf. Investig..

[20] Akan R., Frisk T., Lundberg F., Ohlin H., Johansson U., Li K., Sakdinawat A., Vogt U. (2020). Metal-Assisted Chemical Etching and Electroless Deposition for Fabrication of Hard X-ray Pd/Si Zone Plates. Micromachines.

[21] Chang C., Sakdinawat A. (2014). Ultra-high aspect ratio high-resolution nanofabrication for hard X-ray diffractive optics. Nat. Commun..

[22] Li K., Wojcik M.J., Divan R., Ocola L.E., Shi B., Rosenmann D., Jacobsen C. (2017). Fabrication of hard X-ray zone plates with high aspect ratio using metal-assisted chemical etching. J. Vac. Sci. Technol. B Nanotechnol. Microelectron. Mater. Process. Meas. Phenom..

[23] Li K., Ali S., Wojcik M., De Andrade V., Huang X., Yan H., Chu Y., Nazaretski E., Pattammattel A., Jacobsen C. (2020). Tunable hard X-ray nanofocusing with Fresnel zone plates fabricated using deep etching. Optica.

[24] Romano L., Kagias M., Vila-Comamala J., Jefimovs K., Tseng L.T., Guzenko V.A., Stampanoni M. (2020). Metal assisted chemical etching of silicon in the gas phase: A nanofabrication platform for X-ray optics. Nanoscale Horizons.

[25] Tiberio R.C., Rooks M.J., Chang C., F. Knollenberg C., Dobisz E.A., Sakdinawat A. (2014). Vertical directionality-controlled metal-assisted chemical etching for ultrahigh aspect ratio nanoscale structures. J. Vac. Sci. Technol. B Nanotechnol. Microelectron. Mater. Process. Meas. Phenom..

[26] Kim Y., Tsao A., Lee D.H., Maboudian R. (2013). Solvent-induced formation of unidirectionally curved and tilted Si nanowires during metal-assisted chemical etching. J. Mater. Chem. C.

[27] Romano L., Vila-Comamala J., Jefimovs K., Stampanoni M. (2017). Effect of isopropanol on gold assisted chemical etching of silicon microstructures. Microelectron. Eng..

[28] Chartier C., Bastide S., Lévy-Clément C. (2008). Metal-assisted chemical etching of silicon in HF-H_2_O_2_. Electrochim. Acta.

[29] Cheng S.L., Chung C.H., Lee H.C. (2008). A Study of the Synthesis, Characterization, and Kinetics of Vertical Silicon Nanowire Arrays on (001)Si Substrates. J. Electrochem. Soc..

[30] Kong L., Zhao Y., Dasgupta B., Ren Y., Hippalgaonkar K., Li X., Chim W.K., Chiam S.Y. (2017). Minimizing Isolate Catalyst Motion in Metal-Assisted Chemical Etching for Deep Trenching of Silicon Nanohole Array. ACS Appl. Mater. Interfaces.

[31] Lianto P., Yu S., Wu J., Thompson C.V., Choi W.K. (2012). Vertical etching with isolated catalysts in metal-assisted chemical etching of silicon. Nanoscale.

